# Distinct Hippocampal Oscillation Dynamics in Trace Eyeblink Conditioning Task for Retrieval and Consolidation of Associations

**DOI:** 10.1523/ENEURO.0030-23.2024

**Published:** 2024-04-23

**Authors:** Kayeon Kim, Miriam S. Nokia, Satu Palva

**Affiliations:** ^1^Neuroscience Center, Helsinki Institute of Life Sciences, University of Helsinki, Helsinki FI-00014, Finland; ^2^Department of Neuroscience, Faculty of Health and Medical Science, University of Copenhagen, Copenhagen N DK-2200, Denmark; ^3^Department of Psychology, University of Jyväskylä, Jyväskylä FI-40014, Finland; ^4^ Centre for Cognitive Neuroscience, School of Psychology and Neuroscience, University of Glasgow, Glasgow G12 8QQ, Scotland; ^5^ Division of psychology, VISE, Faculty of Education and Psychology, University of Oulu, Oulu, Ostrobothnia FI-90014, Finland

**Keywords:** classical conditioning, cross-frequency coupling, hippocampus, memory, phase locking

## Abstract

Trace eyeblink conditioning (TEBC) has been widely used to study associative learning in both animals and humans. In this paradigm, conditioned responses (CRs) to conditioned stimuli (CS) serve as a measure for retrieving learned associations between the CS and the unconditioned stimuli (US) within a trial. Memory consolidation, that is, learning over time, can be quantified as an increase in the proportion of CRs across training sessions. However, how hippocampal oscillations differentiate between successful memory retrieval within a session and consolidation across TEBC training sessions remains unknown. To address this question, we recorded local field potentials (LFPs) from the rat dorsal hippocampus during TEBC and investigated hippocampal oscillation dynamics associated with these two functions. We show that transient broadband responses to the CS were correlated with memory consolidation, as indexed by an increase in CRs across TEBC sessions. In contrast, induced alpha (8–10 Hz) and beta (16–20 Hz) band responses were correlated with the successful retrieval of the CS–US association within a session, as indexed by the difference in trials with and without CR.

## Significance Statement

Trace eyeblink conditioning is widely used to study the neural basis of learning. How brain oscillatory signatures for instantaneous retrieval of associations differ from those reflecting long-term memory consolidation is not well understood. We recorded local field potentials from the rat hippocampus during conditioning to dissociate oscillation dynamics associated with these functions. We show that a transient, early, broadband response is correlated with memory consolidation (increase in conditioned responses across training sessions) whereas long-latency sustained alpha and gamma oscillations are associated with the performance within a given trial in a session.

## Introduction

Memories are represented by the distributed activity of neurons organized into neuronal assemblies in hippocampal–neocortical circuits (Squire, 1992; Buzsáki and Moser, 2013). Trace eyeblink conditioning (TEBC) has been widely used as a model system for studying the neural basis of associative learning and memory in a wide range of species from mice to humans. In TEBC, a conditioned stimulus (CS, usually auditory stimulus) is repeatedly paired with an eyeblink-evoking unconditioned stimulus (US, airpuff or electric shock to the eyelid). A successful conditioned response (CR) in response to the CS is dependent on learning, and upon later encounter with the CS, retrieving from memory, of an association between the CS and the US, that is, contingency detection (Prokasy, 1984; Clark and Squire, 1998; Thompson, 2005; Cheng et al., 2008).

Forming an association between two events separated in time is a critical brain function (Pilkiw and Takehara-Nishiuchi, 2018) that depends on the hippocampus (Solomon et al., 1986; Moyer et al., 1990; Weiss et al., 1996; Tseng et al., 2004). Intriguingly, recent work utilizing human intracranial and scalp electroencephalogram (EEG) suggested that the hippocampus could represent both sensory and mnemonic information and act as a switchboard between internal and external representations (Treder et al., 2021). In line, patients with medial temporal lobe (MTL) lesions and memory impairment also exhibited diminished markers for conscious perception (Urgolites et al., 2018) and showed attenuated and temporally more dispersed responses to visual stimuli (Reber et al., 2017), suggesting that hippocampal circuits also represent sensory stimulus properties (Kreiman et al., 2002).

A crucial role in memory formation and the initial encoding of information into neural representations is played by theta (θ, 4–12 Hz) oscillations (for reviews see Buzsáki, 2002; Colgin, 2013, 2016). Hippocampal θ oscillations are abundant during exploratory behavior in rodents, and they organize bits of information into coherent entities (for an example of θ and place cells, see O’Keefe and Recce, 1993). Interestingly, during TEBC, hippocampal θ oscillations increase during the trace period separating the CS offset and the US onset, both in rabbits (Nokia et al., 2009, 2015) and in rats (Nokia et al., 2012). The CS-related θ oscillations in the hippocampus and connected brain structures are thought to reflect the encoding and retrieval of the CS–US contingency (Wikgren et al., 2010; Nokia and Wikgren, 2013; Suter et al., 2019). Additionally, beta (β; 12–30 Hz) and gamma (γ) band (30–40 Hz) oscillations are also prominent in the hippocampus (Colgin et al., 2009), with γ oscillations synchronizing the activity in the hippocampal–entorhinal cortical (EC) loop and relating to encoding and retrieval of associations (Igarashi et al., 2014). Whereas θ oscillations are proposed to allow chunking or grouping of information, β and γ oscillations are proposed to reflect the information per se. In this context, cross-frequency phase–amplitude coupling (PAC) between the θ phase and β/γ oscillation amplitudes is thought to enable the chunking of sensory information (Colgin, 2016).

Despite extensive research on the neural basis of learning using TEBC, the relationship between mapping stimulus contingencies within a trial (encoding and retrieval of the CS–US association) and learning, that is, enforcing their associations across training sessions (memory consolidation), remains unclear. While the role of oscillation dynamics in memory functions is well established, the differences in these dynamics between the formation of mnemonic associations between the CS and US within a trial and memory consolidation of stimulus contingencies across training sessions remains unexplored. With training on the CS–US contingency, the probability of a CR to each CS increases gradually over time. However, even with extensive training, some CS presentations fail to elicit a CR. In this study, we hypothesized that distinct spatiotemporal signatures would characterize mapping CS–US contingencies within sessions (indexed as CR for the trial) and memory consolidation across training sessions (indexed as an increasing proportion of CRs over sessions). To this end, we recorded local field potentials (LFPs) from the dorsal hippocampus in freely moving adult healthy Sprague Dawley male rats during classical TEBC and estimated oscillation dynamics for hippocampal subfields using state-of-the-art analysis approaches.

## Materials and Methods

### Animals

Eight adult healthy male Sprague Dawley rats (Harlan Laboratories/Envigo, weighing ∼300 g, ∼10 weeks) were used as subjects. Food and water were available *ad libitum*, with room temperature and humidity maintained at 21 ± 2°C and 50 ± 10%, respectively. The rats were kept under a 12 h light/dark cycle. All procedures and experiments were conducted during the light cycle. The study was conducted in accordance with Directive 2010/63/EU of the European Parliament and of the Council on the care and use of animals for research. The experiments were approved by the Animal Experiment Board of the Regional State Administrative Agency of Southern Finland. The ARRIVE guidelines (http://arriveguidelines/org/arrive-guidelines) were followed.

### TEBC and analysis of the eyeblink response

**Figure 1.**
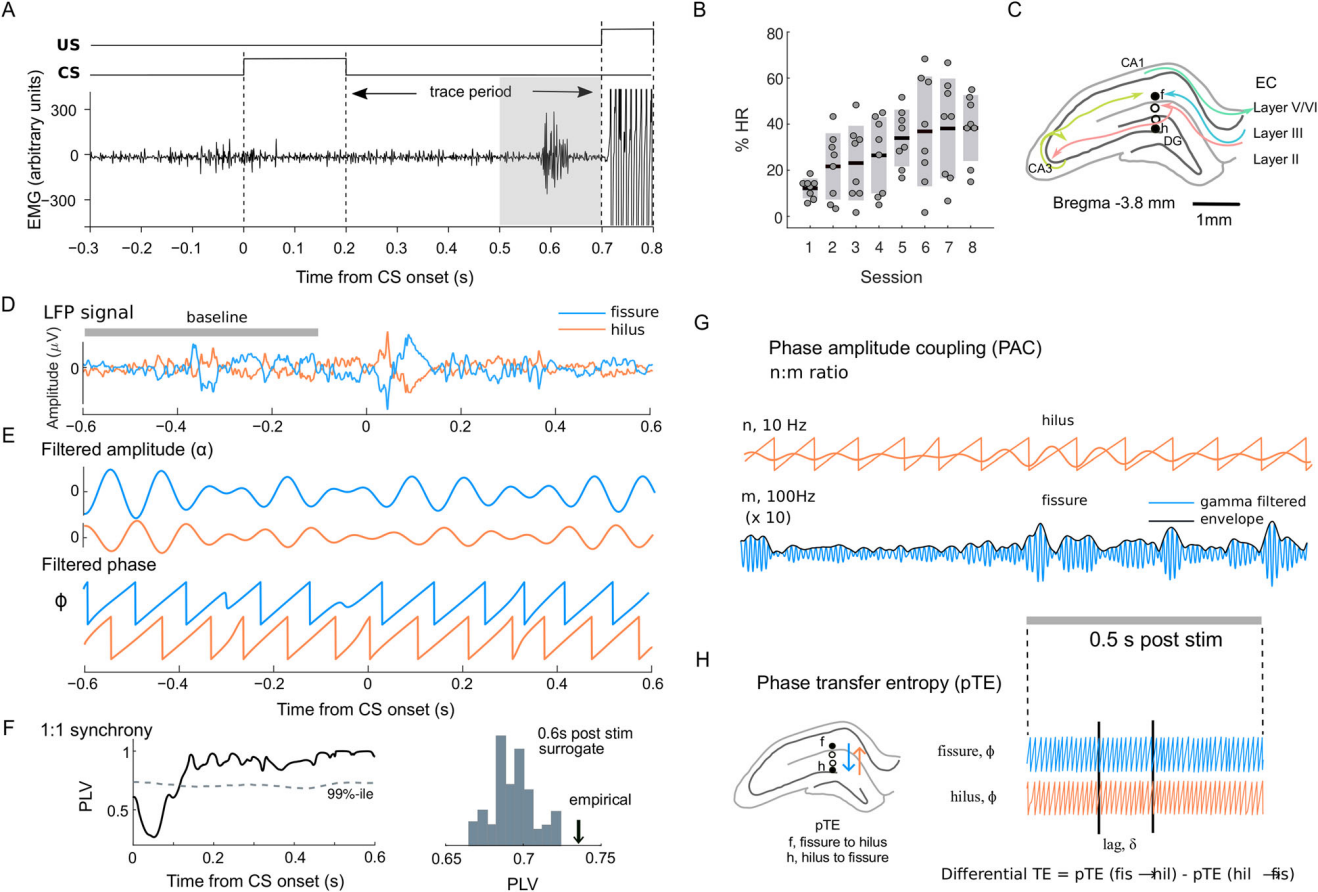
Overview of the approach. ***A***, Task schematics and an example of EMG trace. In the TEBC task, a tone was used as CS followed by the US (periorbital shock). Eyeblinks were recorded with EMG. The gray area indicates the time window when eyeblinks were counted as a CR. ***B***, HR for the CRs as a function of session with mean and SEM. The circles represent data from individual rats (*n* = 8). ***C***, A graphical illustration of recording electrode placement in the rat hippocampus targeting fissure (f, upper) and hilus (h, lower). ***D***, ***E***, An example of a broadband raw signals recorded from fissure (blue) and hilus (orange) for a single trial and of their narrow-band filtering and transformation to complex values to obtain amplitude and phase time series. ***F***, Interareal synchronization between fissure and hilus was estimated by computing PLV between signals and comparing the empirical values to a surrogate distribution. ***G***, The n:m PAC was computed between the phase of a lower frequency (*n*) and amplitude of the higher frequency (*m*). The traces show the θ phase in hilus and the γ amplitude envelope in fissure (black). ***H***, Differential phase transfer entropy (phase dTE) was estimated from the phase-lag information from each region using an analysis window of 0.5 s during the post-CS period. (See Materials and Methods for more details.)

Data were collected during a TEBC task (Fig. 1*A*) using LabVIEW (National Instruments). The animals were conditioned using white noise (75 dB, 200 ms) as a CS and a 100 Hz burst of 0.5 ms bipolar pulses of periorbital shocks (100 ms) as a US. The amplitude of the US was adjusted individually for each animal to elicit a blink response, that is, the unconditioned response in 100% of the trials. Each trial started with the 200 ms CS presentation, followed by a 500 ms stimulus-free trace period, and then a 100 ms shock US. Each animal performed eight sessions, each consisting of 60 trials. Over time, the animals started to blink in response to the CS; that is, they acquired the CR.

Eyeblinks were detected from the EMG signals offline to determine the percentage of CRs (here defined as the hit rate, HR), as in Nokia et al. (2017). In brief, for each trial, the response was defined as a CR if the signal exceeded a threshold of mean + 3 standard deviations (SD) within the last 200 ms of the trace period (Fig. 1*A*). That is, the eyelid started to close immediately before the onset of US. HR was defined as the proportion of trials with a CR (Fig. 1*D*). We then used repeated measures ANOVA (rm ANOVA) to estimate the change in HR across the eight sessions as well as across the animals. Reaction times (RTs) were computed for the trials with CRs.

### Surgery

Rats were anesthetized with an intraperitoneal injection of pentobarbital (60 mg/kg) and treated for pain with carprofen (5 mg/kg, s.c.) and buprenorphine (0.03 mg/kg, s.c.). Using a stereotactic frame, we implanted two four-wire electrodes (Formvar-Insulated Nichrome, bare diameter 50 µm, no. 762000, A-M Systems) chronically to record LFPs from the dorsal hippocampus. The wires were glued together with a tip separation of 200–250 µm. The bundles were implanted with the lowest electrode tip at the dentate gyrus (DG; 3.6–4.5 mm posterior, 1.5–2.2 mm lateral, and 3.6–4.0 mm below bregma; Fig. 1*C*). Skull crews served as the reference (11 mm posterior and 2 mm lateral to bregma) and ground (4 mm anterior and 2 mm lateral to bregma). To stimulate the eyelid and record electromyography (EMG) during TEBC, two bipolar electrodes made of stainless steel wire insulated with Teﬂon (bare diameter 127 µm) were implanted through the upper right eyelid. Finally, the entire construction was secured in place using dental acrylic cement. After the surgery, each rat was allowed to recover for at least 1 week and was medicated for pain with buprenorphine.

### Recordings

A low-noise wired preampliﬁer (10×) was directly attached to the electrode connector in the rat's head. The LFP signals were filtered from 1 to 5,000 Hz, amplified 50×, digitized at 20 kHz, and then low-pass filtered at 500 Hz. Finally, all signals were stored at a sampling rate of 2 kHz (USB-ME-64, Multi Channel Systems).

### Histology

Rats were killed by exposure to a rising concentration of CO_2_ and then decapitated. The locations of the electrode tips in the brain were marked by passing a DC anodal current (200 mA, 5 s) through them. The brain was then removed, fixed in a 4% paraformaldehyde solution, and coronally sectioned with a vibratome (Leica VT1000). The slices were stained with Prussian blue and cresyl violet. The electrode tip locations were determined with the help of a conventional light microscope and a brain atlas (Paxinos and Watson, 1998).

### Signal preprocessing, re-referencing, and filtering

Trials with large LFP fluctuations due to movement or device-related artifacts were excluded if they exceeded 1,500 µV cutoff, resulting in a rejection rate of 0.6%. To remove the stimulus-induced spike artifact, raw signals between −2.5 and 1.5 ms from CS onset and offset were interpolated with an alpha blend fraction of 0.45. Current source density (CSD) profiles were then calculated for re-referencing using the Laplacian re-referencing method with adjacent channels (Mitzdorf, 1985). The signal was then filtered between 3 and 480 Hz using a finite impulse response (FIR) filter with a logarithmically scaled increment of frequency and utilizing a combination of high-pass and low-pass filter pairs (high-pass: 0.6 stop band and low-pass: 1.4 stop band, 60 dB attenuation). The filtered signals were further Hilbert transformed to obtain phase and amplitude time series (Fig. 1*E*).

### Data analysis of oscillation dynamics

We computed local oscillation dynamics using measures of oscillation amplitudes and intertrial coherence (ITC) with a phase-locking factor (J. M. Palva et al., 2005) separately for the hilus and fissure. The amplitude and ITC time series were averaged across trials for each condition from −600 to 600 ms from CS onset for each filtered frequency. The average baseline values from −600 to −100 to CS onset were then subtracted from poststimulus values. Phase time series data were further used to compute interareal synchronization between the fissure and hilus using the phase-locking value (PLV; Fig. 1*F*). PLV was normalized by dividing the value by the averaged PLV value obtained by shuffling trials within a condition and measurement site 100 times to control for the contributions of stimulus-driven artificial synchronization (Hirvonen et al., 2018).

To estimate coupling across the frequencies, PAC was computed with PLV between the phase of the slow oscillation (low frequency, LF) and the phase of the amplitude envelope of the high-frequency (HF) oscillation for the m:n ratios between 2 and 9 (Fig. 1*G*; J. M. Palva et al., 2005; Siebenhühner et al., 2016). Phase transfer entropy (phase TE; Lobier et al., 2014) was computed to identify directionality in the narrow-band oscillatory signals. Phase TE was derived from the instantaneous phase time series of the signal *X*_hilus_(*t*) and *Y*_fissure_(*t*) expressed as *θ*_hilus_(*t*) and *θ*_fissure_(*t*) (Fig. 1*E*). To create a bias-free measure, we computed the differential TE (dTE) derived by pTE_hil→fis_−pTE_fis→hil_ and used this measure for further analysis (Fig. 1*H*).

### Statistical analysis

To avoid different signal-to-noise ratios (SNRs) from influencing the results, the number of trials between conditions (CR vs no-CR; highest HR vs lowest HR) was equalized before statistical analyses by randomly selecting trials to match the minimum number of trials within a session. Statistically significant changes in oscillation amplitudes, ITC, and interareal synchrony were achieved by deriving null distributions (*n* = 20,000) using random flips with a probability of 0.5 (Monte Carlo *p *< 0.025, two-tailed). To correct for multiple comparisons, we used the Benjamini–Hochberg procedure for each analysis time window.

To assess the significant differences between CR and no-CR trials, we first estimated the differences in neuronal dynamics within each animal. We then used a paired *t* test to identify the *t*-threshold (*p *< 0.025, two-tailed) for each time *t* and frequency *f* and obtained the *t*-sum observed value based on temporal adjacency. The maximum *t*-sum observed was tested against the *t*-sum null distribution from 1,000 surrogates (Monte Carlo *p *< 0.05) using cluster-based permutation statistics, which account for multiple comparisons in statistical analysis (Maris and Oostenveld, 2007). The same clustering permutation statistics were performed to compare the sessions with the lowest and highest HR. The null distributions were derived by randomizing trials for comparison between CR and no-CR trials and by randomizing the sessions for comparing learning.

Individual-level statistical analysis for oscillation amplitude differences was computed using a cluster-based permutation method by randomizing the CR and no-CR trials and sessions for the lowest %HR and highest %HR, respectively, to obtain surrogate mean values. Individual-level statistical analysis for ITC values was obtained by comparing each ITC value with a surrogate null distribution [Monte Carlo *p* < 0.05, false discovery rate (FDR) corrected].

To directly study the main effects and the interaction effect of successful memory retrieval within a session (CR vs no-CR) and consolidation across TEBC training sessions, we conducted two-way rm ANOVA on both the oscillation amplitudes and on the ITC within the time-frequency regions of interest (*p *< 0.025). The statistical analysis for 1:1 interareal synchronization between conditions was performed using a two-sample *t* test for the time-frequency regions of interest.

The presence of significant PAC in the poststimulus periods was assessed post hoc using a one-sample *t* test across all subjects and sessions for each pair of low and high frequencies, analysis time windows, and laminar pair (FDR corrected). For statistical analysis of phase TE, surrogate data were obtained by shuffling trials 1,000 times and comparing the surrogate distributions with the empirical data to test the statistical significance against a null hypothesis of no information transfer between the hilus and fissure (Monte Carlo *p *< 0.05, FDR corrected).

The code for phase TE is freely available at the Palva GitHub repository (https://github.com/palvalab/palvalab).

## Results

### Behavioral data

Associative learning was assessed using a TEBC task (Fig. 1*A*, see Materials and Methods for more details). HR (the percentage of CRs) significantly increased as a function of the session (Pearson’s *r* = 0.49; *p *= 4.51 × 10^−5^; Fig. 1*B*), but sessions with the best performance varied across animals. The mean RT for CRs averaged across all sessions and animals was 558.72 ms with an SEM of 5.7 ms. To control the confounding effects of individual differences, we also conducted rm ANOVA, confirming a significant change in HR across sessions (*F*_(7,49) _= 5.72; *p *= 7.17 × 10^−5^) and indicating that the rats learned to anticipate the shock US and shield the eye with a CR as training progressed.

### Spatiotemporal dynamics of oscillations in response to the CS

**Figure 2.**
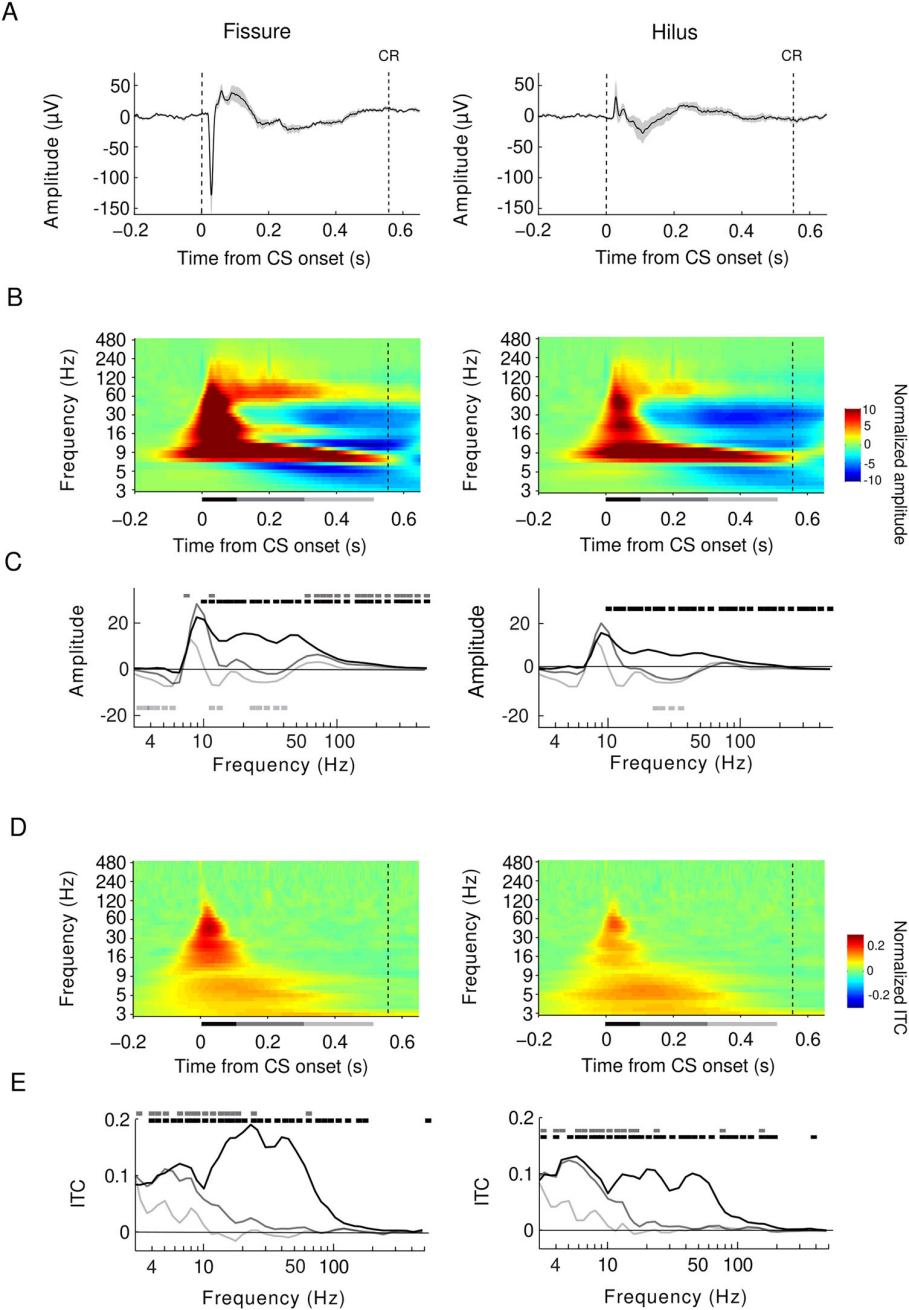
Spatiotemporal dynamics of hippocampal oscillations during TEBC. ***A***, A group-averaged broadband evoked response to the CS, averaged across all trials, sessions, and animals from fissure (left) and hilus (right). Shade indicates SEM. Dashed lines indicate CS onset and mean (±SEM) and latency of CR (558.7 ± 5.7 ms), respectively. ***B***, Baseline-corrected time-frequency representation (TFR) of oscillation amplitudes in response to the CS separately for fissure (left) and hilus (right). Oscillation amplitudes were averaged across trials, sessions, and then across all rats (*n* = 8). The horizontal bar below the panel indicates analysis periods relative to CS onset; 0–0.1 s (black), 0.1–0.3 s (gray), 0.3–0.5 s (light gray). ***C***, Oscillation amplitudes averaged across three time windows. Horizontal colored bars denote statistical significance at *p *< 0.025 (see Materials and Methods for details). ***D***, ***E***, Intertrial coherence (ITC) as estimated with PLF as in ***B*** and ***C***. Horizontal bars denote statistical significance at *p *< 0.025.

Figure 2*A* shows group-averaged CSD re-referenced LFP traces from the hippocampal subfields (fissure, hilus). Consistent with the fissure encompassing dendrites of the DG granule cells receiving the main input from the EC, CS-evoked responses were robust in the fissure but not in the hilus. In addition, the evoked responses were reflected in both oscillation amplitudes and ITC, showing transient broadband responses in the fissure but also in the hilus (Fig. 2*B–E*; Monte Carlo *p *< 0.025, two-tailed, FDR corrected). This time-locked activity was followed by induced alpha [α, 8–12 Hz, note that this band is usually defined as theta (θ) in rodent literature] and high-gamma (high-γ, 50–100 Hz) band responses in the fissure (Fig. 2*B*,*C*, gray, light gray; Monte Carlo *p *< 0.025, two-tailed, FDR corrected). In addition, concurrent suppression of θ (here 4–6 Hz) and β/γ band (23–40 Hz) amplitudes was observed both in the fissure and hilus (Fig. 2*B*,*C*, light gray trace; Monte Carlo *p *< 0.025, two-tailed, FDR corrected). Despite similar spatiotemporal oscillatory profiles in the hilus and fissure, induced amplitudes were stronger in the fissure, which is the primary site of EC inputs via the perforant pathway (Scharfman, 2016).

### Distinct spatiotemporal patterns for the retention of stimulus contingencies between CS–US and consolidation across training sessions

**Figure 3.**
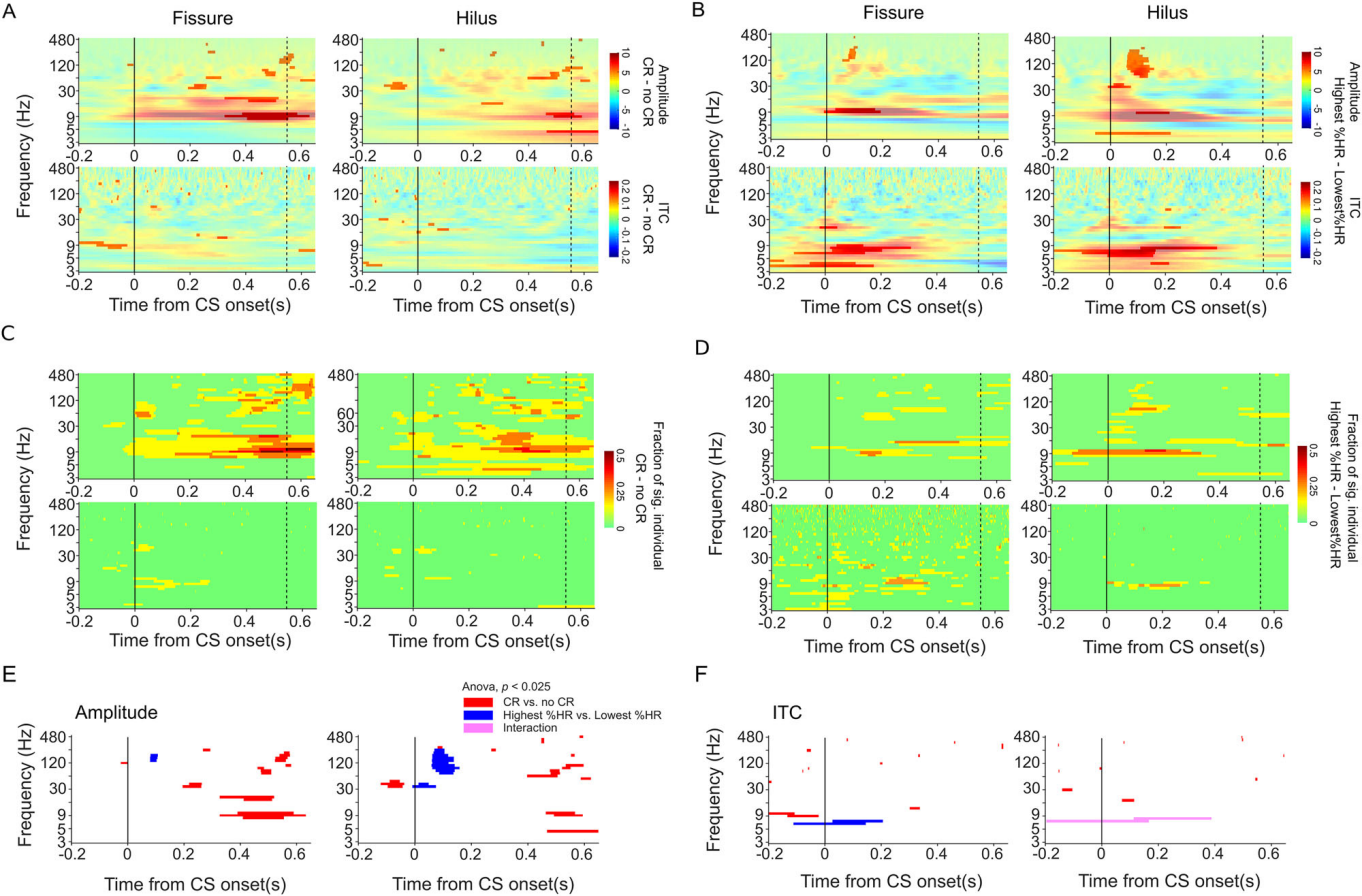
Distinct spatiotemporal profiles of hippocampal oscillations. ***A***, Oscillation amplitudes (top panel) and ITC (bottom panel) for the contrast between CR and no-CR trials. The color scale indicates the amplitude (top) and ITC (bottom) for each time-frequency element. The significant clusters of time and frequency (Monte Carlo *p *< 0.05) are highlighted with transparency while nonsignificant observations are nontransparent. Dashed lines indicate mean RT. Solid lines indicate CS onset. ***B***, The same as in ***A***, but for the contrast between sessions with the lowest and the highest HR. ***C***, Individual level statistics for the contrast between CR and no CR and for ***D***. The lowest versus highest HR. Color scale indicates the fraction of individuals that show significance in each time-frequency element (Monte Carlo *p *< 0.050). ***E***, ***F***, Two-way ANOVA. ***E***, The representation of significant time and frequencies denoting the main effects of CR versus no-CR (in red) and Highest %HR versus Lowest %HR (in blue), along with the interaction of the two factors (in pink) on amplitude (ANOVA *p *< 0.025). Fissure (left), hilus (right). Black lines denote CS onset ***F***. The same convention as in ***E*** but illustrates the main effects of the ANOVA factors on ITC.

We next explored whether distinct spatiotemporal patterns associated with the retrieval of stimulus contingencies between the CS and the US and with memory consolidation across the daily training sessions could be identified (Fig. 3*A–D*). To examine this, we utilized the eyeblink–CR as an approximation of the contingency detection and retrieval of the CS–US association, comparing trials with CR to those without CR (=no CR) within the session. After the initial trials during which representations of the CS and US are formed, CR is thought to depend on retrieving the association between the CS and the US as well as planning and executing the subsequent motor response (Prokasy, 1984). To assess the spatiotemporal patterns of oscillations related to successful contingency detection, we computed oscillation amplitudes and ITC separately for trials with and without CR for each animal and condition. Sustained amplitudes in α and β bands at ∼300 ms from the CS onset were stronger for the CR trials than for the no-CR trials in both the fissure and to a lesser extent in the hilus (Fig. 3*A*; Monte Carlo *p* < 0.050). In contrast, there was no noticeable difference between the CR and no-CR trials in the ITC (Fig. 3*A*, bottom). In addition to group-level significance, the results were further reproduced by performing statistical analysis at the individual level (Fig. 3*C*; Monte Carlo *p* < 0.050).

We then tested whether we could dissociate a separate spatiotemporal pattern for predicting learning across the daily training sessions by comparing the data from the session with the highest and lowest %HR for each animal. Transient broadband amplitudes and ITC were stronger for the session with the highest %HR than for the lowest %HR (Fig. 3*B*; Monte Carlo *p* < 0.050). The results were further reproduced by performing statistical analysis at the individual level (Fig. 3*D*; Monte Carlo *p *< 0.050).

Next, we performed an rm ANOVA to probe whether the different oscillatory patterns associated with the CR and no-CR trials and memory consolidation across sessions in the previous analysis were statistically significantly different. This analysis confirmed that sustained α and β band amplitudes were stronger for the CR versus no-CR difference (α, *F*_(1,127) _= 25.94, *p *= 1.44 × 10^−6^; β, *F*_(1,127) _= 13.12, *p *= 4.40 × 10^−4^) than those for the memory consolidation across sessions in the fissure (α, *F*_(7,127) _= 1.19, *p *= 0.32; β, *F*_(7,127)_ = 0.88, *p *= 0.53; Fig. 3*E*, left panel). Similar trends were observed in the hilus (α, CR vs no-CR, *F*_(1,127)_ = 10.1, *p *= 0.002; memory consolidation, *F*_(7,127)_ = 1.39, *p *= 0.21; Fig. 3*E*, right panel).

In contrast, the transient γ band amplitude response was stronger for memory consolidation (fissure, *F*_(1,31)_ = 8.23, *p *= 0.008; hilus, *F*_(1,31)_ = 13.17, *p *= 0.001; Fig. 3*E*) than that for CR vs no-CR (fissure, *F*_(1,31)_ = 4.23 × 10^−4^, *p *= 0.98; hilus, *F*_(1,31)_ = 0.87, *p *= 0.36), whereas there were no significant effects in the lower frequencies despite the statistical differences shown in Figure 3*B*. However, the transient α band ITC exhibited a stronger association with memory consolidation across TEBC sessions in the fissure (*F*_(1,31)_ = 10.75; *p* = 0.003) compared with that in CR vs no-CR trials (*F*_(1,31)_ = 0.24; *p* = 0.63; Fig. 3*F*, left panel). An interaction effect was observed between retrieval of the CS–US contingency and memory consolidation across the daily training sessions in the hilus (*F*_(1,31)_ = 10.85; *p* = 0.003; Fig. 3*F*, right panel). This analysis confirmed that the retrieval of stimulus contingencies between the CS and the US and memory consolidation across the daily training sessions are associated with distinct spatiotemporal patterns of neuronal oscillations.

### Phase synchronization and directional interactions among the hippocampal subfields

**Figure 4.**
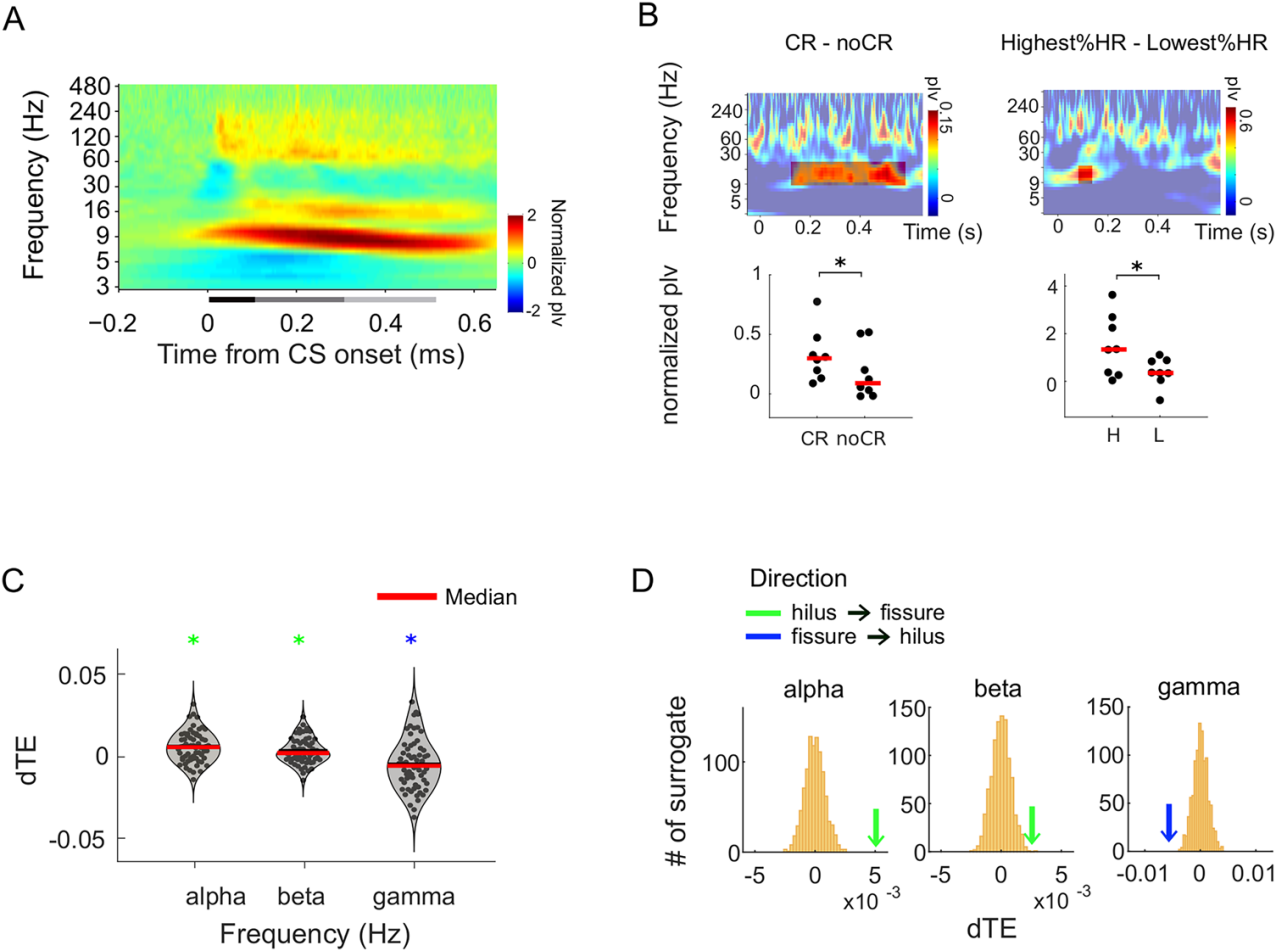
Interareal synchronization and directed interactions between hilus and fissure. ***A***, Group-averaged interareal synchronization between hilus and fissure estimated with PLV. Data are averaged across trials, sessions, and then across all rats (left). ***B***, Same as in ***A*** but presenting PLV for the contrast between CR and no-CR trials (left) and the contrast between the highest and the lowest HR (right). The transparency highlights the time and frequencies with a significant difference. The scatter plots below show individual rat data, with red horizontal lines indicate the median, (paired *t* test, *p *< 0.05). ***C***, Group-averaged differential phase transfer entropy (dTE) across sessions and rats during a 0.5 s post-CS period. Green marks indicate significant dTE from hilus to fissure, blue mark indicates significant dTE from fissure to hilus. ***D***, Distribution of mean dTE derived from surrogate data by trial shuffling. The green or blue arrows in each subplot indicate the mean dTE of the empirical data.

In the hippocampus, information flows from the EC to the dendrites of the DG granule cells and CA1 pyramidal cells lining the fissure (perforant path) and from the DG to CA3 pyramidal cells via the hilus (mossy fibers). From the hilus, information feeds back to the CA1 pyramidal cell dendrites lining the fissure (Fig. 1*C*). To study the information flow between the hippocampal subfields, we computed the phase synchronization of oscillations between the hilus and fissure. Robust phase synchrony between the hilus and fissure was found in the α band and in the high-γ band (Fig. 4*A*, right; Monte Carlo *p *< 0.010, FDR corrected) despite the lack of concurrent oscillation amplitude increases. Interestingly, for local oscillations, sustained α-β band (9–16 Hz) phase synchronization between the hilus and fissure was significantly higher for the highest %HR versus lowest %HR (Fig. 4*B*, right; *p *< 0.05), whereas the early transient α and β band phase synchronization was stronger for CR versus no-CR trials (Fig. 4*B*, left; *p* < 0.05).

To further establish whether oscillatory interactions would be directional as predicted by the feed-forward information flow from the fissure to the hilus or feedback processes from the hilus to the fissure, we estimated the directionality of coupling using pTE (Lobier et al., 2014), focusing on the frequencies showing significant interareal synchrony. Differential TE (dTE) was derived by the difference between pTE_hil→fis_ and pTE_fis l→hil_ across all sessions and rats. In the α and β bands, information flow was significant from the hilus to the fissure (Fig. 4*C*,*D*, green marks; Monte Carlo *p *< 0.050), whereas oscillations at high-γ (80 Hz) band showed the opposite direction, from the fissure to the hilus (Fig. 4*C*,*D*, blue marks; Monte Carlo *p *< 0.050).

### Nested α-γ oscillations associated with learning across sessions

**Figure 5.**
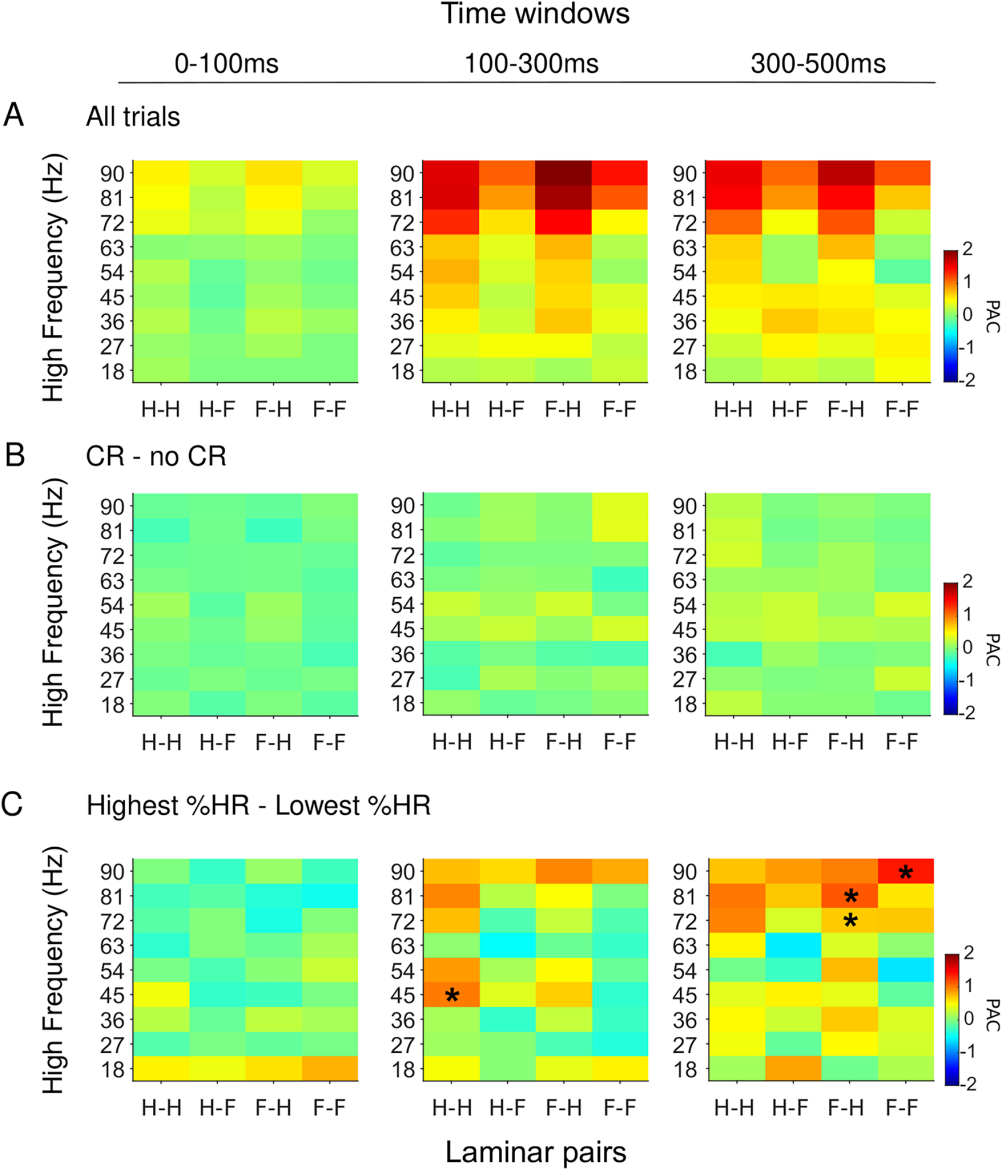
PAC for CS. PAC on each laminar pair (H, hilus; F, fissure) represented in the *x*-axis, and the high-frequency is plotted in the *y*-axis. Each panel shows each analysis window of interest (rows from left to right; leftmost, 0–0.1 s; middle, 0.1–0.3 s; rightmost, 0.3–0.5 s after stimulus onset). ***A***, PAC using all trials, PAC difference between poststimulus and prestimulus time windows. ***B***, PAC difference between CR and no-CR trials. ***C***, PAC difference between highest %HR and lowest %HR. Black stars indicate statistically significant difference between the contrasting conditions (*p *< 0.05, FDR corrected).

We then examined the PAC between oscillations across frequencies and laminar pairs. Specifically, we focused on the coupling of α phase (9 Hz) with higher frequencies. Robust α:γ PAC characterized the LFP signal within the hilus and between the hilus and fissure across all windows (Fig. 5*A*). PAC did not differ between CR and no-CR trials (Fig. 5*B*); however, there was a notable increase in α:γ PAC during sessions with the highest %HR compared with the lowest %HR sessions between 100 and 500 ms from CS onset (Fig. 5*C*; paired *t* test; *p* < 0.05, corrected), showing that α:γ strengthens as a function of learning.

## Discussion

TEBC has been widely used to study neuronal mechanisms of learning in humans (Cason, 1922), rabbits (Schneiderman et al., 1962), and rodents (Weiss et al., 1996; McEchron and Disterhoft, 1999; Tseng et al., 2004). In this study, we used a classical TEBC task in rats to investigate hippocampal oscillation dynamics linked to the retrieval of stimulus contingencies within a trial (approximates as CR) from those dynamics associated with memory consolidation, that is, the improvement in performance across the training sessions. Our results demonstrate that different spatiotemporal patterns of hippocampal oscillations reflect these functions. Transient CS time-locked responses were correlated with memory consolidation (learning across sessions), but not with the within-session retrieval of stimulus contingencies. In contrast, induced θ/α and γ band oscillations at later time windows were correlated with the successful retrieval of the CS–US contingency within sessions, but not with memory consolidation across the training sessions. These results demonstrate that learning subprocesses are associated with distinct oscillation dynamics.

### Transient early broadband activity predicts learning across sessions

The early transient broadband response was similar to that found in the sensory cortices in humans for perception (S. Palva et al., 2005; Palva et al., 2011; Hirvonen and Palva, 2016; Julku et al., 2021) and short-term memory encoding (Palva et al., 2011), as well as in the primary auditory cortex in guinea pigs (Voigt et al., 2018). The transient γ band amplitude response, and θ-α ITC, predicted memory consolidation (improved performance) across the training sessions. This result agrees with previous findings of θ band phase at CS onset predicting hippocampal responses and learning in TEBC in rabbits (Seager et al., 2002; Nokia et al., 2015) and in human episodic memory tasks (Griffiths et al., 2021). The early latency of this transient response suggests that it might reflect the encoding of the CS features and feed-forward processing of sensory information (Lamme and Roelfsema, 2000), rather than short-term memory of the CS, retrieving the CS–US association from memory, or the motor response planning. This supports the idea that improved behavioral performance across the training sessions is due to strengthened stimulus representations of CS, which leads to memory consolidation over time. This is also supported by the interaction effect between memory consolidation and contingency detection in the hilus.

### Within-trial retrieval of stimulus contingencies is associated with sustained oscillations

Beyond the encoding of the physical environment, such as spatial mapping during navigation or exploration (O’Keefe and Dostrovsky, 1971; Morgan et al., 2011), the hippocampus is also implicated in representing and encoding visual information in primates (Lee et al., 2012; Jutras et al., 2013; Zeidman et al., 2015). In this study, sustained α and γ band responses were correlated with successful retrieval of CS–US contingencies, but not with learning across daily training sessions. This indicates that processes related to retrieving the CS–US association are distinct from those mediating improvements in learned behavior over extended periods (hours, days). Our study dissociating these two processes in a classical TEBC task provides a novel view of the role of concurrent θ and γ oscillations, where the traditional view has been that θ band amplitudes (Berry and Thompson, 1978; Winson, 1978) and θ band phase (Nokia et al., 2015) are related to learning. Our study demonstrates that these oscillations are, in fact, associated with the successful retrieval of stimulus contingencies. Taken together, we propose that the switch from transient broadband oscillations to sustained θ, α, and γ oscillations reflects a transition from unconscious learning-induced feed-forward processing to short-term mnemonic associations between CS and US, enabling the retrieval of stimulus contingencies and appropriate action selection.

### Interareal coupling between the hippocampal subregions

Interareal synchronization between fissure and hilus in the α, β, and γ bands is consistent with the well-established anatomical connectivity between hippocampal subregions (Andersen et al., 2007) and narrow-band synchronization among hippocampal areas (Gloveli et al., 1997; Akam et al., 2012). Similar to the temporal pattern observed in local oscillation amplitudes, the transient early α−β band synchronization was correlated with memory consolidation across the training sessions, whereas the later sustained α−β band synchronization was correlated with the retrieval of CS–US contingencies. In these frequency bands, the direction of information flow was significant from the hilus to the fissure, while the opposite was observed for high-γ activity. Considering that the neocortex sends projections to DG granule cells, whose dendrites occupy the molecular layer along the fissure, and that the DG granule cells convey the signal to CA3 pyramidal cells via their axons passing in the hilus, these results suggest that, in accordance with previous work (Jensen et al., 2015; Michalareas et al., 2016; Richter et al., 2017), oscillation-based interactions in the γ band could mediate feed-forward processing of information (along the granule cells), while the slower frequencies could exert feedback from EC to DG granule cell and CA1 pyramidal cell dendrites.

### Nested alpha–gamma coupling predicts memory consolidation

In accordance with θ oscillations yielding clocking mechanisms for the temporal coding of information in the hippocampal–subicular network (Buzsáki and Chrobak, 1995; Chrobak and Buzsáki, 1996; Lisman and Jensen, 2013), robust PAC between the phase of α oscillation and the amplitude envelope of γ oscillation characterized local and interareal hippocampal oscillation dynamics. Intriguingly, similar to the interareal synchronization, the strength of this α:γ PAC predicted memory consolidation across training sessions, suggesting that learning over time is dependent on a complex pattern of both local and interareal interactions between hippocampal subregions. These data agree with prior studies that have observed θ–γ PAC in the rat hippocampus during a T-maze task (Tort et al., 2008) and associative learning (Tort et al., 2009), as well as in the neocortex during a recognition memory task (Siebenhühner et al., 2016; Bahramisharif et al., 2018).

## Synthesis

Reviewing Editor: Niko Busch, Westfalische Wilhelms-Universitat Munster Decisions are customarily a result of the Reviewing Editor and the peer reviewers coming together and discussing their recommendations until a consensus is reached. When revisions are invited, a fact-based synthesis statement explaining their decision and outlining what is needed to prepare a revision will be listed below. The following reviewer(s) agreed to reveal their identity: Peter Steinmetz, Scott Brincat. Note: If this manuscript was transferred from JNeurosci and a decision was made to accept the manuscript without peer review, a brief statement to this effect will instead be what is listed below.

# Synthesis

Thank you for revising the manuscript and addressing the reviewers' comments. The original reviewers and I have evaluated the revised manuscript accordingly. Both reviewers agree that many of their concerns have been successfully addressed. Their remaining comments concern two issues: questions about the design of the statistical analysis and requests for further language editing. I encourage you to address each comment carefully.

1. The statistical analysis included two tests for each dependent variable: with-CR vs. no-CR (i.e., within-session effect) and highest-HR vs. lowest-HR (i.e., across-sessions effect). Only one test was found to be significant for each dependent variable. For instance, alpha and beta/gamma power was stronger for with-CR compared to no-CR trials, but did not show a significant difference in the comparison of highest-HR vs. lowest-HR sessions. The analysis did not determine whether the two contrasts were significantly different. In other words, is the within-session effect on oscillatory power actually significantly stronger than the between-session effect? This question cannot be addressed using two separate pairwise tests; testing for a significant interaction using a 2 × 2 factorial design would be required.

This point is elaborated in detail in:

Nieuwenhuis, S., Forstmann, B. U., & Wagenmakers, E. J. (2011). Erroneous analyses of interactions in neuroscience: a problem of significance. Nature neuroscience, 14(9), 1105-1107.

In my opinion, the abstract and discussion use correct phrasing of the statistical result because they make no claims that one effect is *stronger* than the other. However, many readers may miss this nuance. I see two possible ways to address this problem. These effects can be tested in a way that allows for a direct comparison between the two contrasts, for instance, using a factorial design. Alternatively, given that the wording seems to reflect a correct interpretation of the results, you could also explicitly emphasize that the analysis, strictly speaking, does not show that the within-session effect is stronger or weaker than the across-session effect.

2. Both reviewers pointed out that the quality of writing could be further improved. Please address the specific issues raised by them, and I also recommend scrutinizing especially the revised sections of the manuscript. I agree with the reviewer that some of these sentences are difficult to comprehend.

3. In principle, eNeuro permits citations of unpublished preprints. However, the preprint by Treder et al. (2021) is published in PNAS. I recommend replacing the BioRxiv preprint with the peer reviewed publication.

# Reviewer 1

In this second revision of this paper the authors have now clarified the contrast they are trying to highlight. That is a difference in oscillatory signatures between the between session increase in the rate of CRs and the within session changes in the rate of CRs. Unfortunately, the paper performs no direct comparison between the results due to these two types of learning. While the authors show that one type of oscillation is associated with within session changes and another associated with between session changes, it is unclear whether each type of oscillation might be associated with both types of changes. Since this is the main contrast noted in the title and highlighted in the abstract, these differences need to be directly and clearly demonstrated. A direct test of the oscillatory amplitudes between the trials and the sessions would seem to be in order.

My pain level in reading the english in this paper is slightly lower than with the prior version, but the use of english is still so poor as to be inappropriate for a scientific article published in english. The lack of proper pronouns and conjunctions makes this paper extremely difficult to parse. I would strongly suggest that the authors hire a native english speaker to edit the paper; while their efforts to improve the use of english are admirable, it really is not sufficient here. Unfortunately the authors have chosen not to substantially clarify the english by making it more concrete and have also failed to provide a high level clear explanation of the overall design of the contrasts here.

The following are just a few examples of the sort of poor use of english which makes this paper painful to read for a native speaker:

p 2, l 26 "is not well understood"

p 3 l49 Does the journal permit citing preprints?

p 3 l49 "In line, " is not a proper way to transition here. order is wrong in 'exhibited also'

p 4 l74 The sentence starting "Related, " is properly begun and run on to an extraordinary extent.

p10 l226 In this section I would expect to see directly the contrast between the oscillatory activites which comprise the early transient broadband activity and the long latency changes correlated with performance. This contrast is not clear here.

# Reviewer 2

I think the writing has greatly improved in the latest revision, and the manuscript is much clearer in its aims now. I have only a few minor comments and suggested changes/fixes to the writing below.

Minor comments

-----------------------

General: I think "memory retrieval" is a reasonable characterization of the CR vs no-CR contrast, but I would clarify it as *successful* memory retrieval.

Intro: I think it would be helpful to somewhere - perhaps as a first sentence in the paragraph starting on line 71 - clarify that CRs remain somewhat probabilistic even after extended training, eg:

"With training on the CS-US contingency, the probability of a CR to each CS increases gradually over time. But even with extensive training, some CS presentations fail to elicit a CR."

Line 270: Why is "θ" in the section title? Shouldn't this just be something like "α : γ"?

Fig. 3 A,B - On my copy at least, these look weird in the latest submission - it looks like only the significance masking is plotted, but not the actual underlying data. However, this figure otherwise looks identical to the previous submission, so I've assumed the figure is unchanged, and this is just a formatting glitch(?). If this was an intentional change, the authors should revert to the old version or another format where the actual underlying data (not just clusters of significance) is shown.

Fig. 4B - Unclear why significance is depicted differently here than in the previous figure (rectangular outline vs transparency mask), or how their significance test could label a precisely rectangular region.

Fig. 4D might be clarified a bit by labeling the plots with which parts correspond to the hilus to fissure direction (positive values) vs the fissure to hilus direction (negative values).

Fig. 5 - I feel it's a bit confusing to label phase-amplitude coupling as "PLV" here. Of course, the method used must be explained in Methods, but in the text and figure legends, I'd suggest just referring to phase-amplitude coupling as "PAC".

Typos / Suggested language fixes

-------------------------------------------

Line 14: replace "the" with "successful"

Line 20: insert "successful" before retrieval

Line 24: insert "the" before "brain"

Line 25: replace "for" with "reflecting successful"

Line 30: replace "the" with "successful"

Line 49: Add "with that idea" after "In line"

Line 73: change "enforcing" to "strengthening"

Line 74: change "related" to "relatedly"

Line 282: change "stimulus contingencies" to "stimulus-response contingencies"?

Line 283: change "approximates as" to "measured by a successful"
